# Self-structuring in Zr_1−x_Al_x_N films as a function of composition and growth temperature

**DOI:** 10.1038/s41598-018-34279-w

**Published:** 2018-11-05

**Authors:** N. Ghafoor, I. Petrov, D. Holec, G. Greczynski, J. Palisaitis, P. O. A. Persson, L. Hultman, J. Birch

**Affiliations:** 1 Thin Film Physics Division, Department of Physics, Chemistry, and Biology (IFM), Linköping University, Linköping, SE-581 83 Sweden; 2 Department of Physical Metallurgy and Materials Testing, Montanuniversität Leoben, Franz-Josef-Strasse 18, A-8700 Leoben, Austria; 3 Materials Science Department, and Frederick Seitz Materials Research Laboratory, University of Illinois, 104 S. Goodwin Avenue, Urbana, Illinois 61801 USA

## Abstract

Nanostructure formation via surface-diffusion-mediated segregation of ZrN and AlN in Zr_1−x_Al_x_N films during high mobility growth conditions is investigated for 0 ≤ × ≤ 1. The large immiscibility combined with interfacial surface and strain energy balance resulted in a hard nanolabyrinthine lamellar structure with well-defined (semi) coherent c-ZrN and w-AlN domains of sub-nm to ~4 nm in 0.2 ≤ × ≤ 0.4 films, as controlled by atom mobility. For high AlN contents (x > 0.49) Al-rich ZrN domains attain wurtzite structure within fine equiaxed nanocomposite wurtzite lattice. Slow diffusion in wurtzite films points towards crystal structure dependent driving force for decomposition. The findings of unlikelihood of iso-structural decomposition in c-Zr_1−x_Al_x_N, and stability of w-Zr_1−x_Al_x_N (in large × films) is complemented with first principles calculations.

## Introduction

The finding of high-temperature age-hardening in B1-cubic(c) Ti_1−x_Al_x_N thin films via isostructural spinodal decomposition^1–3^ have steered research towards understanding of self-organized nanostructuring in the wider family of transition metal (TM) aluminum nitride materials. The prospects are to tailor coherency strain and elastic modulus difference between the nanosized domains to advance functional hard coatings with tailored properties including superhardness, toughness, thermal stability, ultrawear resistance, and corrosion protection.

The self-organization is a result of large miscibility gaps for the TMN-AlN pseudobinary systems, where large positive enthalpy of mixing favors the segregation of the binaries. ZrN-AlN system has one of the largest positive enthalpies of mixing among the transition metal (namely: Ti, Sc, Cr, Zr, Hf) aluminum nitrides^4,5^. The chemical driving force for iso-structural decomposition, H_mix_, is theoretically predicted to be twice as large in c-Zr_1−x_Al_x_N as compared to c-Ti_1−x_Al_x_N, around the metastable c-AlN solubility limit which is as low as x~0.45 in c-Zr_1−x_Al_x_N^6^. Experimentally, metastable c-Zr_1−x_Al_x_N (x < 0.43) alloys are formed during low temperature physical vapor deposition processes^7–9^, however, spinodal decomposition at elevated temperatures has not been evident as for Ti_1−x_Al_x_N. For instance, coherent c-AlN domain formation is not observed at any stage during decomposition in X-ray/electron diffraction or lattice resolved imaging. Sheng. *et al*.^10^ have predicted that due to relatively large lattice mismatch between the fcc ZrN and the fcc AlN, the coherent spinodal decomposition is hindered due to concurrent rapid phase transformation from c-AlN to the stable hexagonal(h) AlN(B4,wurtzite).This condition needs experimental testing, as provided herein.

Regardless of synthesis and decomposition route, the cubic alloys have shown high-temperature age-hardening and mechanical stability up to 1250 °C^7,9,11^. Instead of coherency strain generation during spinodal decomposition, the observed hardness increase is either attributed to the formation of strained nanometer sized domains accompanied with lattice distortion in sputtered c-Zr_0.57_Al0_0.43_N films^11^, or defect recovery of c-ZrAlN domains in arc evaporated Zr_0.44_Al_0.56_N_1.20_ films^7^. In this work, we establish both through growth experiments and first principle calculations that spinodal decomposition is unlikely in cubic ZrAlN alloys.

Another scenario for the inevitable decomposition process is via non-isostructural phase formation i.e., c-Zr_1−x_Al_x_N directly decomposes into c-ZrN and h-AlN domains. The mechanism is *spinodal-like* and the coherency strain, generated due to formation of semi coherent nano-domains, results in higher hardness. Experimentally, the decomposition into two phase coherent nanostructures and associated hardness increase has been assumed first by Sanjinés *et al*.^11^ and later by Mayrhofer *et al*.^9^ in annealing experiments of sputtered c-Zr_1−x_Al_x_N films. We, on the same account, have shown a highly regular nanostructure of cubic and hexagonal intergrown semi-coherent single-crystal phases evolved during high-temperature magnetron sputter thin film synthesis of Zr_0.64_Al_0.36_N^12^. In this case, the self-organization is surface driven and the synergistic result of kinetic limitations, where the enthalpy reduction balances both investments in interfacial surface and strain energies. Here, the non-isostructural decomposition is further investigated in terms of nanostructure ordering within a given temperature-composition window and mechanical strength of the composites.

As a further extension, we address the origin of driving force for phase separation in h-Zr_1−x_Al_x_N alloys, a subject which requires corresponding insight. Similar to cubic phase alloys, calculations suggest positive mixing enthalpies for hexagonal B_k_ and wurtzite(w) B4 alloys^6^. The metastable w- Zr_1−x_Al_x_N (x = 0.46 and 0.7) in cathodic arc evaporated films was found to phase separate through spinodal decomposition, resulting in nanoscale compositional modulations of AlN- and ZrN-rich layers forming within the hexagonal ZrAlN lattice^13,14^. Stable crystal structure after 1100 °C and slow coarsening of the nanodomains in subsequent decomposition in these films are in remarkable contrast to the metastable c-ZrAlN. In this work we expound the self-segregation mechanisms in hexagonal phase alloys and pinpoint underlying thermodynamics of such processes.

While common practice is to study the decomposition mechanisms in post-annealing experiments, we present surface driven self-organization *during* ion assisted magnetron sputter deposition of Zr_1−x_Al_x_N films grown on to single-crystal and polycrystalline substrates. To discern the segregation route and phase evolution, single-phase cubic/hexagonal and fully-segregated two-phase films are deposited with various compositions and growth temperatures. We present a comprehensive structural and chemical analysis and resolve the mechanisms governing the formation of diverse nanostructures over the entire compositional range. Particularly, the driving force to self-organize nanostructures in cubic and hexagonal systems is discussed backed by first principles calculations. The relationship between nanostructures and mechanical strength of the films is established.

## Experimental Details

Two series of Zr_1−x_Al_x_N films were deposited on 10 × 10 mm^2^ substrates; MgO (001), MgO (111), and polished polycrystalline WC + Co. In total seven Zr_1−x_Al_x_N films with x = 0, 0.03, 0.2, 0.36, 0.49, 0.75, and 1, were deposited in *Series-1* at the substrate temperature (T_s_) of 800 °C, chosen to activate surface diffusion for the system. In *Series-2*, T_s_ was varied between 500–900 °C and five films were deposited with a nominal composition of Zr_0.69_Al_0.31_N, chosen for the nitride alloy to be inside the miscibility gap. For cross-sectional TEM analyses, these last five layers were also deposited in a single stacked multilayer structure, where temperature was decreased every 30 minutes from 900 °C to 500 °C in steps of 100 °C. The substrates were cleaned in ultrasonic bath with trichloroethylene, acetone and 2-propanol, respectively and were blown dried in N_2_ before mounting in the substrate holder which was designed to place three substrates simultaneously. The substrate temperature was measured through thermocouple placed directly under the heater and pre-calibrated for ZrN film on MgO(001) using pyrometer.

The choice of MgO(001) was made as it shares the B1 NaCl structure with ZrN (a = 4.58 Å) with a nominal in-plane lattice mismatch of 8.7%. The optimization of the deposition process for instance, choice of Ar(4 mTorr)/N_2_(0.5 mTorr) ratio and substrate temperature of 800 °C were made to obtain good quality epitaxial ZrN films up to 0.5 μm thickness on MgO(001), and conditions were kept constants for MgO(111) and WC-Co substrates for all x ≠ 0 films. For a complementary structural analysis, the MgO(111) substrate was chosen which matches a hexagonal plane with the B4 wurtzite structure of AlN (a = 3.11, c = 4.98 Å) with in-plane lattice mismatch of 4.4%. The depositions on polycrystalline WC-Co substrates were made to differentiate the epitaxial effects of substrates on the texture and phase formation.

Depositions were carried out in a high vacuum dc unbalanced magnetron sputtering system with maintained base pressure of about 3 × 10^−7^ Torr aided by a loadlock transfer system^15^. For reactive sputtering, 75 mm diameter Zr (99.9%) and Al (99.999%) targets, mounted off-axis forming an angle of 25*°* with the substrate normal, were used in Ar/N_2_ atmosphere at a total pressure of 4.5 mTorr and target power p_Zr_ + p_Al_ = 250 W. The rotating substrate was ~12 cm far from the targets. Desired compositional range 0 ≤ x ≤ 1 to deposit *Series-1* was then achieved by changing individual powers between 0–250 W. All single films were deposited for 1.5 h yielding 1–1.5 µm thick films suitable for nanohardness and structural analysis. Prior to igniting the plasma and adjusting the desired T_s_ the substrates were degassed at 900 °C for ~1 hr. For obtaining good quality films, a high flux of low energy ion bombardment of the sputtering gas ions was utilized, and substrates were biased to −30 V. The details of deposition chamber and magnetic field configuration for availability of high ion flux near the vicinity of growing films is described elsewhere^15^.

Time-of-flight elastic recoil detection analysis (TOF-ERDA) was used to determine Zr:Al ratio and level of impurities in the films, using a 40 MeV ^127^I^9+^ beam at 67.5° incidence relative to the surface normal and a 45° recoil angle^16^. The data were evaluated off-line with the CONTES code^17^. N 1 s, Al 2p, and Zr 3d X-ray Photoelectron Spectroscopy (XPS) core-level spectra were acquired to analyze the chemical bonding structure of Zr and Al atoms within the nanostructures using an Axis Ultra DLD spectrometer operating at a base pressure of 1.5 × 10^−7^ Pa with monochromatic Al Kα radiation (hν = 1486.6 eV). In order to avoid uncertainties related to commonly used binding energy scale referencing against C 1 s line of adventitious carbon^18^, spectra were aligned to the Fermi level cut-off. The spectra were acquired from the 0.3 × 0.7 mm^2^ area centered at 3 × 3 mm^2^ portion of the sample previously sputter-etched with 0.5 keV Ar^+^ ions incident at the angle of 70° from surface normal. Deconvolution and quantification was performed using the Casa XPS software applying the manufacturer’s (Kratos Analytical Ltd.) sensitivity factors^19^.

The coupled ω-2θ X-ray scattering analysis was made using Philips X’Pert-MRD operating with Cu-K_α_ radiation with configuration of crossed slits (2 × 2 mm^2^) as primary optics and parallel plate collimator (0.27°) with flat graphite crystal monochromator as secondary optics. Film hardness was determined using a UMIS nanoindentor equipped with Berkovich diamond tip of approximately 150 nm radius. To determine the average hardness approximately 20 indents were performed on each sample with a 9 mN load, selected to avoid substrate effects and to obtain load independent mechanical response. The unloading segments of each indent were analyzed to extract hardness and reduced modulus following the approach of Oliver and Pharr^20^.

Several S/TEM platforms equipped with conventional imaging and analytical tools were extensively used in this work. A FEI Tecnai G2 TF 20 UT FEG microscope in micro and nanoprobe mode provided overview and high resolution TEM and scanning TEM (STEM) images. An FEI instrument Tecnai Orisis with a combination of a high brightness field emitter and windowless EDX detection using Silicon Drift Detector (SDD) technology with a significantly improved sensitivity for light elements was used for EDX elemental analysis. A double-corrected Linköping FEI Titan^3^ 60–300 (Hillsbro, OR, United states) operated at 300 kV was also used for STEM imaging. Electron transparent cross-sectional and plan-view TEM specimens were prepared by conventional mechanical polishing and ion thinning techniques.

## Calculation Methods

First principles calculations were performed using the Vienna Ab initio Simulation Package^21,22^ employing the Projector Augmented Wave pseudopotentials^23^ for describing the electron-ion interactions. The quantum-mechanical electron-electron interactions were described with the Generalised Gradient Approximation as parametrised by Perdew and Wang^24^. The used plane wave cut-off energy of 500 eV and reciprocal space sampling with 15 × 15 × 15 and 6 × 6 × 4 k-meshes for the cubic and wurtzite unit cells, provide total energy accuracy of approximately 1 meV/at^6^.

The compositional disorder was modelled using Special Quasi-random Structures^25^ of 3 × 3 × 2 cubic (36 atoms) and 2 × 2 × 2 wurtzite (32 atoms) unit cells^26^. Each cell was fully relaxed, i.e., the total energy was minimised with respect to specific volume, unit cell shape and atomic positions. The convergence criterion was set to 10^−5^ eV and 10^−4^ eV for the electronic and ionic loop, respectively. The further reported energies for the hexagonal phase correspond to either B4 or B_k_ structure yielding lower energy for a given composition (for more details, see ref.^6^). The mixing enthalpy, *H*_mix_, of the quasi-binary Zr_1−*x*_Al_*x*_N systems was calculated as

1 $${H}_{{\rm{mix}}}={E}_{{\rm{tot}}}(\xi  \mbox{-} {{\rm{Zr}}}_{1 \mbox{-} {\rm{x}}}{{\rm{Al}}}_{{\rm{x}}}{\rm{N}},{{\rm{V}}}_{\xi })-[(1\mbox{--}x){E}_{{\rm{tot}}}({\rm{\alpha }} \mbox{-} {\rm{ZrN}},{{\rm{V}}}_{\alpha })+x{{\rm{E}}}_{{\rm{tot}}}({\rm{\beta }} \mbox{-} {\rm{AlN}},{{\rm{V}}}_{{\rm{\beta }}})],$$

where α, β, and ξ are cubic or hexagonal, and *E*_tot_(X, *V*) is the total energy (per atom) of a phase X at specific volume *V*. We note that the isostructural decomposition corresponds to the same phases for all ξ, α, and β, and, unless states otherwise, the respective volumes are equilibrium volumes corresponding to pressure p = 0. The individual calculated total energy, specific volume and bulk modulus data points were fitted with third (energy) and second (volume, bulk modulus) order polynomials of the composition, *x*.

## Results

### Composition and deposition parameters

TOF-ERDA results are listed in Table 1 for the measured films in the two series. The analysis revealed a negligible level of residual gas impurities like carbon, oxygen, and sputtering gas argon comprising less than 3 at.% of the film composition. In the S*eries-1* with varied Zr:Al ratio, all films except AlN_0.9_ (x = 1) are stoichiometric with respect to nitrogen, within the experimental uncertainty, which means that N content is measured up to 50 ± 2 at.%. The under-stoichiometric AlN_0.9_ film contains relatively higher amount of oxygen, probably due to a reduced system gettering effect in the absence of Zr sputtering. In the films deposited in *Series-2* with varying substrate temperature, Al concentration of ~15 at.% seems to be independent of T_s_, whereas Zr slightly increases and nitrogen decreases with increasing T_s_ from 500 to 900 °C. In the Zr_0.69_Al_0.31_N_0.97_ film grown at 900 °C slightly higher amount of carbon and oxygen impurities are measured which can be a result of degassing inside the high vacuum chamber at elevated heating.

**Table 1:** Chemical composition and deposition temperature of monolithic films measured by ERDA.

Sample ID	Temp. (°C)	Zr (at.%)	Al (at.%)	N (at.%)	C (at.%)	O (at.%)	Ar (at.%)	Composition
***Series-1***								
1	800	50	0	49	0.8	0.2	0	ZrN_0.98_
2	800	49.7	1.7	48.2	0.2	0.2	0	Zr_0.97_Al_0.03_N_0.94_
3	800	40.3	10	48.3	0.4	0.9	0.1	Zr_0.80_Al_0.20_N_0.96_
4	800	32	18	48.4	0.2	0.9	0.5	Zr_0.64_Al_0.36_N_0.97_
5	800	24.2	23	49.2	0.2	0.4	0.3	Zr_0.51_Al_0.49_N_1.04_
6	800	12.1	36.2	49.3	0.2	0.3	0.2	Zr_0.25_Al_0.75_N_1.02_
7	800	0	51	45.7	0.3	3	0	AlN_0.9_
***Series-2***								
1	500	32.1	15.4	51.4	0.4	0.4	0.3	Zr_0.68_Al_0.32_N_1.08_
2	600	32.1	15.1	51.2	0.6	0.6	0.4	Zr_0.68_Al_0.32_N_1.08_
3	700	32.9	15	50.3	0.5	0.4	0.9	Zr_0.69_Al_0.31_N_1.05_
4	800	33.3	15.3	49.3	0.6	0.4	1.1	Zr_0.69_Al_0.31_N_1.01_
5	900	34	15.1	47.6	1.4	1.7	0.2	Zr_0.69_Al_0.31_N_0.97_

The analysis of target power ratio versus Zr:Al metal content in the films revealed slightly higher sputter rate of Al compared to Zr at given power in the films. The results are plotted in supplementary material in Fig. SM1 together with an optical photograph of the corresponding 1.5 μm thick films on MgO(001) substrates deposited in *Series-1* at 800 °C. The color change from light-gold to golden, brown, and transparent upon increasing the AlN content is due to changes in electronic band structure as investigated by Lamni *et al*.^27^.

### Structural characterization of the films

**Figure 1:**
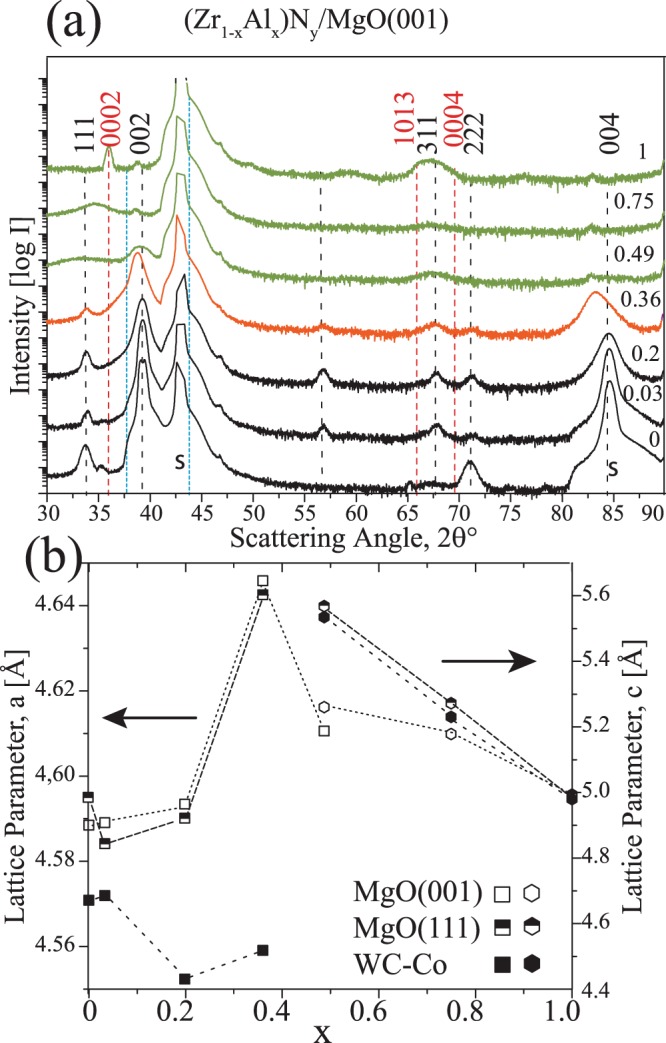
(**a**) ω-2θ X-ray scans of Zr_1−x_Al_x_N_y_ (0 ≤ x ≤ 1) films deposited on MgO(001) substrate *in Series-1*.The scans are offset vertically and labelled according to Al content, x, in the films. The dotted lines are marked at standard c- ZrN (black), c-AlN (blue), and h- AlN(red) lattice reflections. (**b**) lattice parameter extracted from c-002 and/or w- 0002 reflections for all the films deposited in *Series-1:* shown in (**a**) and Fig. SM2.

Figure 1(a) shows ω-2θ scans from Zr_1−x_Al_x_N films deposited in *Series 1* on MgO(001) substrate. The diffraction data of simultaneously deposited films on MgO(111) and WC-Co substrates is shown in the supplementary material in Fig. SM2. The results show that ZrN(x = 0) film on MgO(001) exhibit near epitaxial growth with very low intensity 111 and 222 peaks from c-ZrN. For this, more than a micrometer thick film, FWHMs of ZrN 002 peak is measured to Γ_2_θ = 0.046° and Γ_ω_ = 0.279°. Since, no 111 orientation was seen in 0.5 μm thick ZrN films deposited during the optimization process, we infer that competitive texture evolution occurs at later stage in thicker films under the present growth conditions. Upon adding Al, a decrease in intensity with increasing FWHM of 002 and 004 is observed simultaneous to appearance of 022 and 311 peaks of the cubic phase in Zr_0.97_Al_0.03_N_0.94_ and Zr_0.80_Al_0.20_N_0.96_ films. The scans from films with x = 0, 0.03, and 0.2 are shown in black to categorize films showing peaks from single cubic phase. The film Zr_0.64_Al_0.36_N_0.96_ with next higher Al content, also shows single cubic phase with less intense and broader peaks. However, the peaks are shifted towards lower 2θ values (0.45° shift of 002 reflection) and for separating this feature, the scan is drawn in different color (orange). A very alike peak shift is observed for 0.5 μm thick Hf_1−x_Al_x_ N/MgO (001) film with x = 0.54 when grown at 600 °C^28^ and Zr_1−x_Al_x_ N/Si(001) film with x = 0.52 grown at 500 °C^6^. The significant peak broadening in combination with peak shift indicates that simultaneous to loss of crystallinity a second phase appears in the films and this could be an onset of non-isostructural decomposition as discussed further with the TEM images.

In high-AlN-containing films i.e., x = 0.49, 0.75, and 1, cubic diffraction peaks gradually disappear and instead 0002 and 1013 peaks corresponding to wurtzite phase appear. A low intensity broad wurtzite peak centered at 33.06° in x = 0.49 film, becomes stronger and moves to higher 2θ towards w-AlN in Zr_0.25_Al_0.75_N_1.02_ and AlN_0.9_. The wurtzite phase containing film scans are drawn in green.

Due to epitaxy the ZrN(x = 0) film deposited on MgO(111) substrate exhibit strong (111) texture, which becomes weaker upon adding 3 and 20 at.% Al [see Fig. SM2(a)] The films with high Al content (x = 0.49, 0.75, and 1) grown on MgO(111) show higher intensity, smaller FWHM, and slightly smaller 2θ angle of w-0002 reflection in x ≠ 1 films. The latter can be a consequence of a higher substitution of Zr in the wurtzite lattice. The XRD of films deposited on polycrystalline WC-Co substrate [Fig. SM2(b)] reveal that (111) texture is dominant in ZrN(x = 0) film and the preferential orientation completely switches from (111) to (002) upon adding 20 at.% Al. The films with x = 0.49–1 show identical features as are observed for MgO substrates. The lattice parameter calculated from the peak positions either/or of c-002 and w-0002 reflections is plotted as a function of x in Fig. 1(b) for the three substrates. The sharp increase in out-of-plane cubic lattice parameter for x = 0.36 on MgO grown films and change in wurtzite lattice with increasing Al is evident in this illustration.

**Figure 2:**
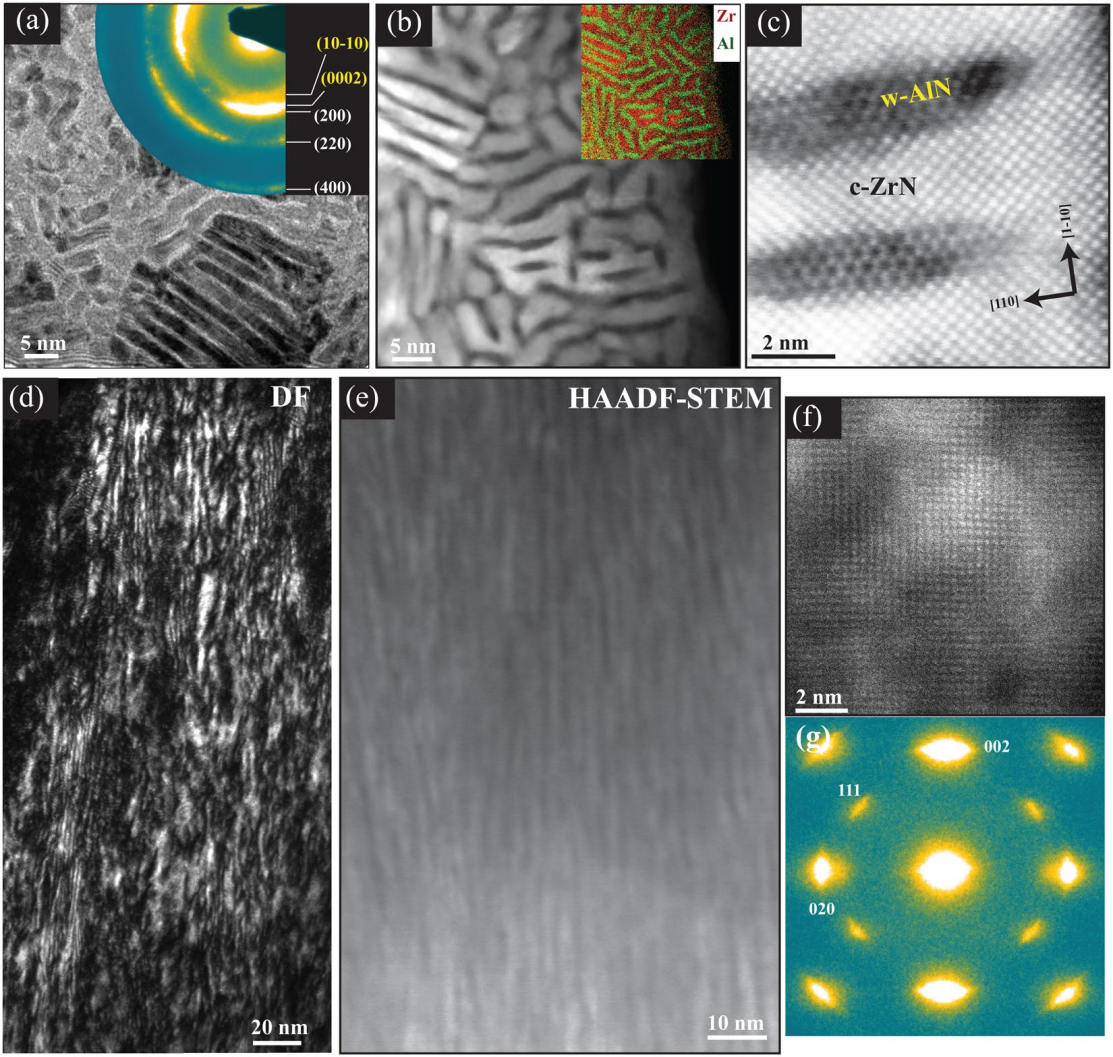
Plan-view (**a**–**c**) and cross-sectional (**d**–**g**) TEM images of Zr_0.64_Al_0.36_N/MgO(001). (**a**) Overview BF-TEM and corresponding SAED, (**b**) HAADF-STEM image with corresponding EDX map. (**d**) Overview DF-TEM, (**e**) HAADF-STEM, (**c**,**f**) HR-HAADF-STEM, (**g**) SAED. For both orientations electron beam was aligned parallel to ZrN[001] zone axis.

According to XRD, the transition from dominant cubic phase films to wurtzite phase containing films occurs in the range 0.2 < x < 0.5. A significant feature is an abrupt increase in the out-of-plane lattice parameter i.e., ZrN 002 peak shifts towards lower angles, e.g., for x = 0.36 film. To further analyze the origin of peak shift, Zr_0.64_Al_0.36_N_0.97_/MgO(001) film was examined both in plan and cross-sectional view by transmission electron microscopy. The TEM/STEM images, SAED, and EDX map in plan-view, shown in Fig. 2(a–c), reveal that about 2–3 nm wide ZrN rich platelets are segregated by ~1.5 nm wide AlN containing phase, and that the AlN-rich platelets attain wurtzite lattice while the ZrN-rich platelets attain cubic structure. The area imaged in aberration corrected STEM micrographs (in a-c) is assured to be well aligned so that electron beam is parallel to <001> zone axis of the lamellae. The topotaxial relationship between the lamellae is found to be $(110)ZrN//(11\bar{2}0)AlN\,and$ $[1\bar{1}0]\,ZrN//[2\overline{11}0]AlN.$

In cross-sectional analysis, the method more in practice to characterize film-substrate morphology, the conventional diffraction contrast dark field (DF) TEM image of Zr_0.64_Al_0.36_N/MgO(001) in Fig. 2(d), reveals a dense crystalline film structure comprising nano-sized columns aligned in the growth direction. Knowing the platelet appearance in plan-view images, the nano-columns are termed *lamellae* here in this article. A Z-contrast HAADF-STEM image in Fig. 2(e) distinctly shows that the film is heterogeneous and bright/dark lamellar contrast corresponds to ZrN-/AlN-rich segregation, respectively. However, unlike two phase lattices observed in plan-view images, high-resolution lattice-resolved X-STEM image in Fig. 2(f) reveals semi-coherent cubic lattice across bright and dark contrast regions. The atomic planes often tend to bend or distort while crossing the AlN-rich areas. Appearance of (111) spots in SAED pattern in (g), obtained along <001> zone axis from the larger film area, reflects in-plane rotation of B1-cubic crystalline lamellae having 002 planes parallel to the substrate, giving rise to a fibre texture. Also, no obvious signs of the hexagonal phase, except for faint broad intensity inside c-020 reflections is observed in this orientation of the film.

**Figure 3:**
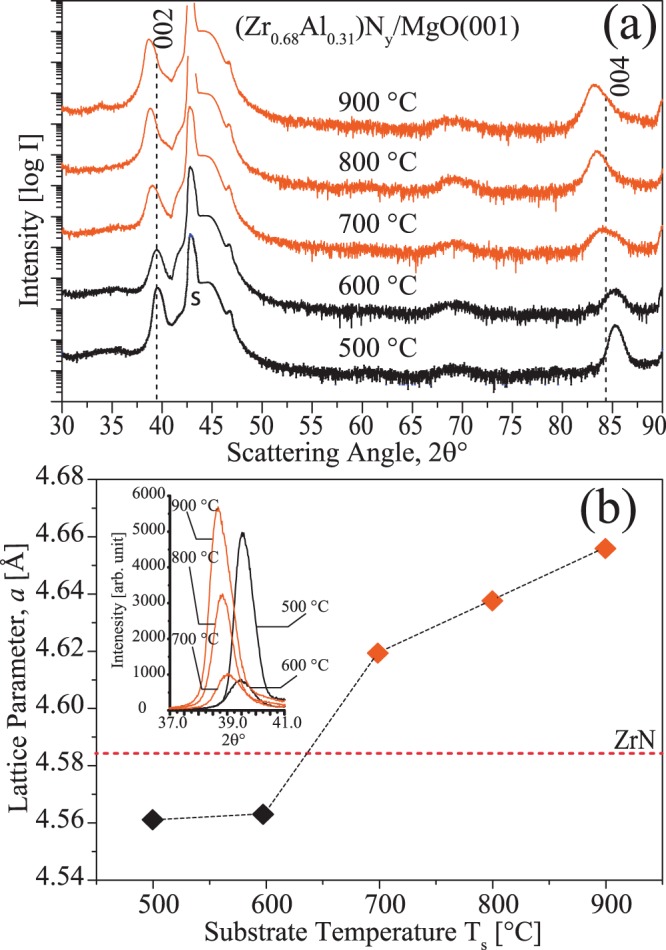
(**a**) ω-2θ X-ray scans of series-2; Zr_0.68_Al_0.31_N/MgO(001) films deposited at different T_s_. The intensity is plotted on log-scale and for clarity scans are vertically offset. The substrate peaks are labeled with s. (**b**) Lattice parameter extracted from c-002 peak positions (the inset shows the intensity comparison of 002 peak on a linear scale). Orange color indicates film with large lattice parameter than ZrN.

Since the self-organization into nano-lamellar structure occurs during the growth, the mechanism can be considered as surface initiated, and the impact of growth temperature should thus be determined. This is explored for dual phase films with nominal composition of Zr_0.69_Al_0.31_N grown on MgO(001) at different temperatures in *Series-2* (see Table 1 for composition detail). The ω-2θ XRD scans in Fig. 3(a) show that when grown between 500–900 °C all films exhibit only two peaks corresponding to 002 and 004 diffraction planes of B1 phase. The intensity comparison of 002 peak in Fig. 3(b) (scans from 2θ = 37–41° plotted on a linear scale without offset) reveal that film grown at 500 °C exhibit high intensity and small FWHM. The intensity dramatically decreases and FWHM increases for the 600 °C film. The change in lattice parameter, *a*, extracted from 002 peak position as a function of T_s_ is plotted in Fig. 3(b). The decrease in *a* for both 500 and 600 °C grown films is indicative of solid solution formation for x = 0.31 and in agreement with calculations^6,10^. At 700 °C, low intensity peaks shift to lower 2θ values, identical to what is observed for x = 0.36 film grown at 800 °C in *Series-1*. The lattice parameter keeps increasing for 800 and 900 °C films up to 0.466 nm, with the accompanied decrease in FWHM of the peaks. The 900 °C film exhibits higher intensity than the 500 °C sample with comparable FWHM. Like in Fig. 1, the profiles of films with lattice parameter larger than standard ZrN are colored in orange.

**Figure 4:**
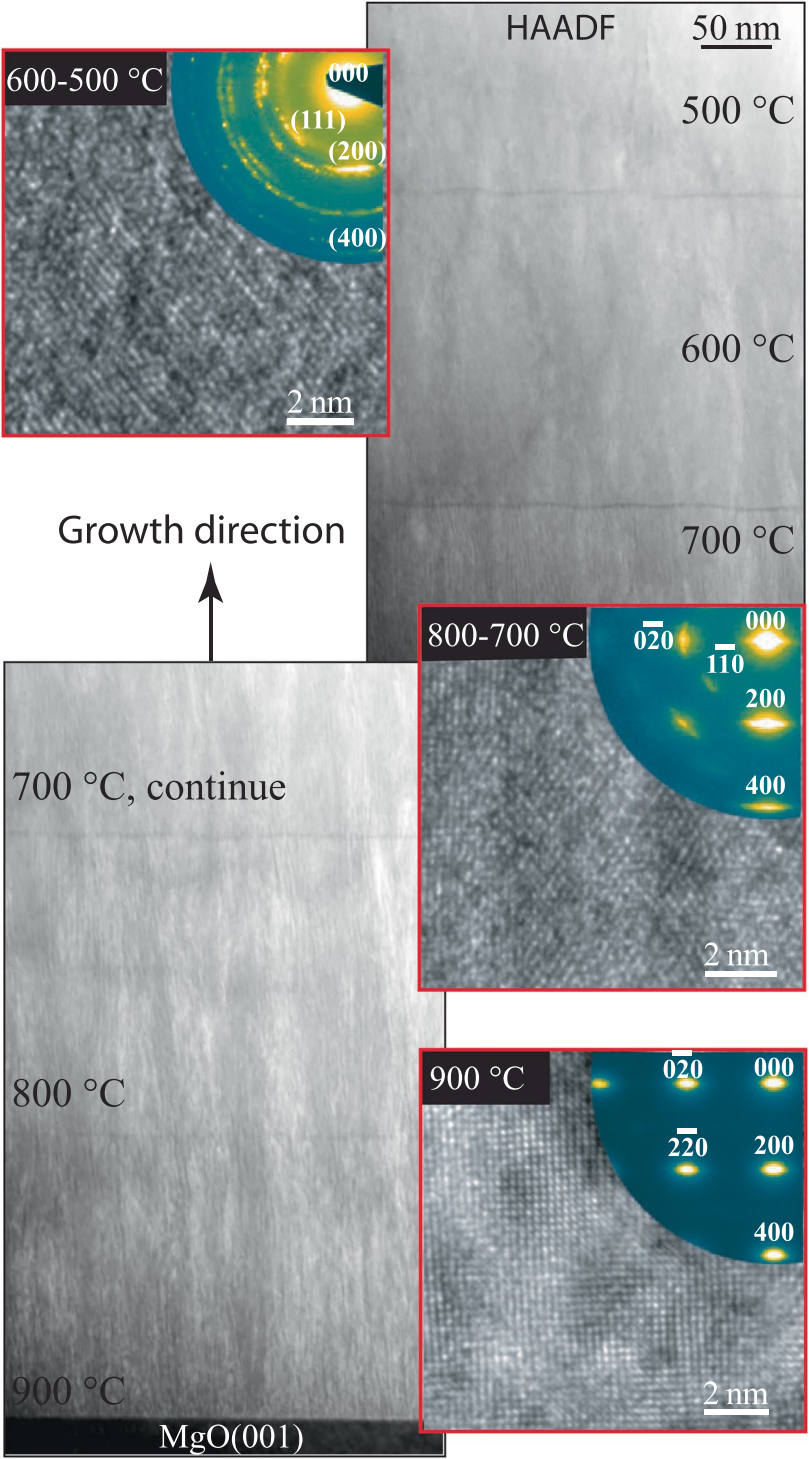
Cross-sectional STEM images of stack of five layers grown on MgO(001) at T_s_ values decreasing from 900 to 500 °C by 100 °C with nominal compositions of (Zr_0.64_Al_0.36_)N_._ The insets (in red) are representative HRTEM images and SAED patterns of the layers.

In order to elucidate the effect of surface diffusion on the phase separation in Zr_0.69_Al_0.31_N, a five-layer stack structure in which substrate temperature during deposition was varied for each layer from 900 °C (bottom) to 500 °C (top) in steps of 100 °C is studied in cross-sectional TEM shown in Fig. 4. In overview STEM images, the dense nano lamellar structure similar to the one described in Fig. 2, is apparent in the multilayer deposited at 900 °C and 800 °C. The lamellae are also present in the multilayer deposited at 700 °C, but lamellae’s width is narrower than the bottom layers. The contrast in the image clearly shows that upon lowering the temperature to less than 600 °C, the lamellar morphology of segregated phases abruptly switches to more homogeneous and uniform elemental distribution. The lattice-resolved TEM image and SAED pattern of the 900 °C layer show coherent cubic lattice for both ZrN and AlN lamellas in <100> projection. Due to topotaxial crystallographic relationship, the lamellas are not distinct in cross-section image obtained along <100> zone axis. The in-plane elongation of diffraction spots, arranged in B1 single-crystal fashion in SAED pattern, reflects limited width of lamellas. For 800 and 700 °C films, the lamellae grow with in-plane rotation creating a fibre texture which made 111 spots to appear in SAED pattern. Thus, the lamellae are projected in <110> and because of in-plane rotation, lamellar structure is feebly visible in the respective lattice resolved images. No obvious diffraction from wurtzite crystal structure is detectable in SAEDs obtained along <100> zone axis from 900–700 °C layers. The SAED pattern as well as lattice resolved images of multilayers deposited at 600 and 500 °C revealed more of a polycrystalline-like growth with weak 002 texture in the growth direction in agreement with XRD.

**Figure 5:**
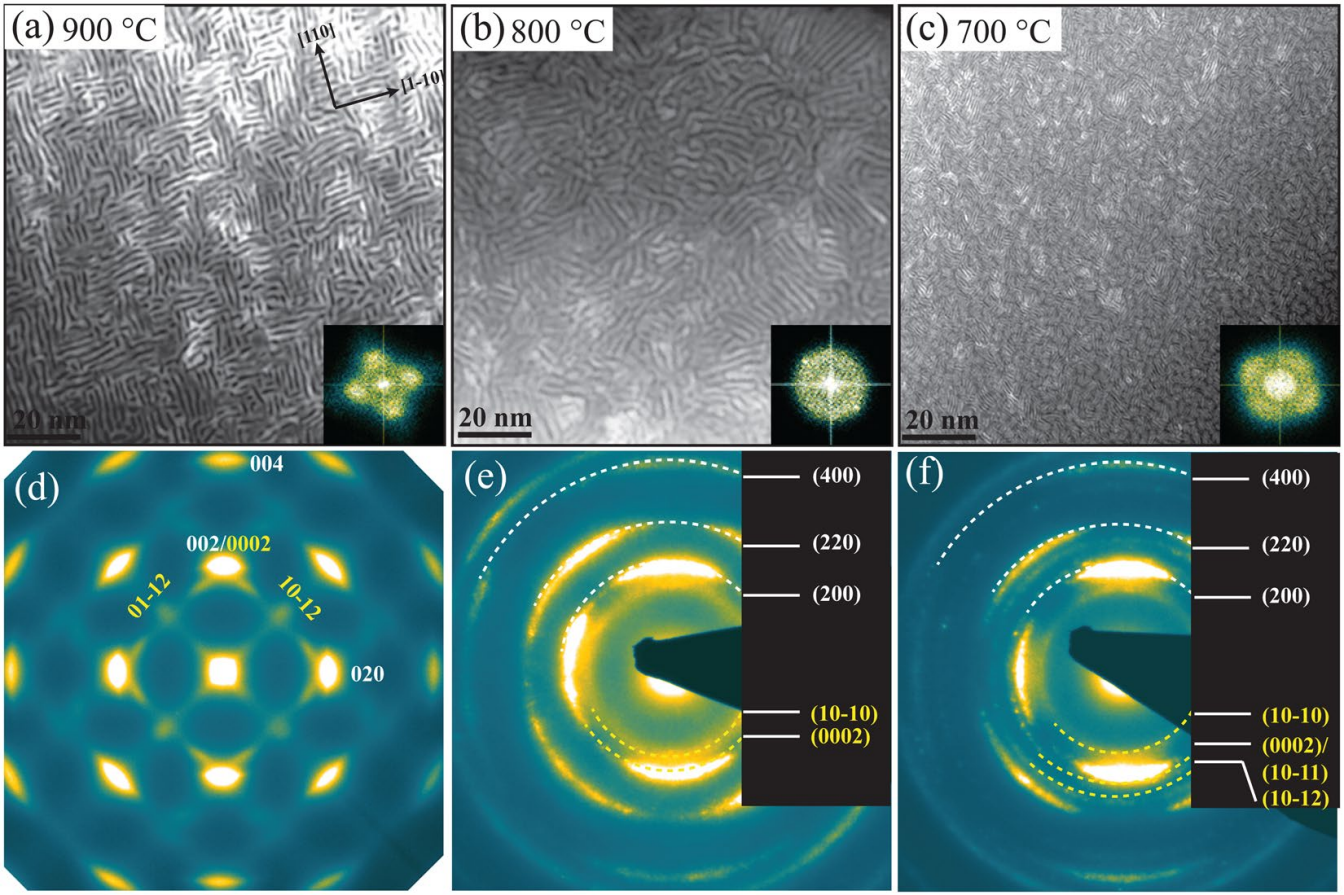
(**a**–**c**) Plan-view HAADF-STEM images of (Zr_0.69_Al_0.31_)N/MgO(001) films grown at 900, 800, and 700 °C, respectively. Insets are magnified FFT around (000) reflection. (**d**–**f**) corresponding SAED patterns.

To further comprehend growth temperature influence on self-structuring HAADF-STEM images and SAED patterns were recorded in plan-view, for 900 °C, 800 °C, and 700 °C deposited Zr_0.69_Al_0.31_N films. The images are shown in Fig. 5(a–f), respectively. The nanostructure in the films grown at 900 and 800 °C consists of AlN-rich platelets (dark contrast) with a hexagonal crystal structure interweaved as a labyrinth by ZrN-rich platelets (bright contrast) with an fcc crystal structure. Due to striking resemblance of this two-phase nanocomposite to a labyrinth, structure is named as *“nanolabyrinth*”. The growth mechanisms and crystallography of nanolabyrinthine film has been previously reported by combining HAADF-STEM imaging in plan-view, synchrotron-based pole figures, and atom probe tomography^12,29^. The local topotaxial relationship between the phases is found to be $(110)ZrN//(11\bar{2}0)AlN\,and\,[1\bar{1}0]\,ZrN//[2\bar{1}\bar{1}0]\,AlN$ in both films shown in Fig. 5(a,b) [also shown in Fig. 2(e–g)]. However, the 900 °C-grown sample exhibits long-range order of labyrinth orientation to about several square *μ*m, compared to 800 °C grown sample where order extends only to 20–30 square nm. The ordering length is also apparent in the diffraction patterns. The long-range highly-ordered labyrinth in the growth plane in 900 °C grown sample results in distinct spots pattern including both cubic and wurtzite phases (for details see^12^). The diffraction arcs from the wurtzite phase are clearly visible in 800 °C film in this alignment compared to cross-sectional view in Fig. 4. The compositional modulation and ordering length is also apparent in the FFT’s of the STEM images. The nanostructure of 700 °C grown film is composed of segregated ZrN and AlN small needle-like platelets appeared as sub-nm thin lamellae in Fig. 4 in the cross-sectional view. The FFT shows that structure is more ordered compared to 800 °C film. This extremely fine nanocomposite structure resembles a knitted sheet from a thread composed of two different fibres. The corresponding SAED shows diffraction arcs indicative of presence of both cubic and wurtzite phases. It is an important finding as this, together with lattice resolved image analysis and XRD, indicates that AlN rich domains attain wurtzite-crystal structure upon segregation regardless of domain size and no c-AlN forms at any stage.

So far, results indicate that at growth temperatures above 700 °C and Al content of 31–36 at.%, ZrN and AlN segregate into cubic and wurtzite phase, respectively, and their topotaxial relationship leads to a self-assembled nanolabyrinth structure. Also, upon formation of w-AlN in-plane compression in ZrN lattice results 002 peaks in XRD to shift towards lower 2θ angles. While c-ZrAlN solid solutions are present for T_s_ ≤ 600°, no c-AlN during the self-assembly process is found.

**Figure 6:**
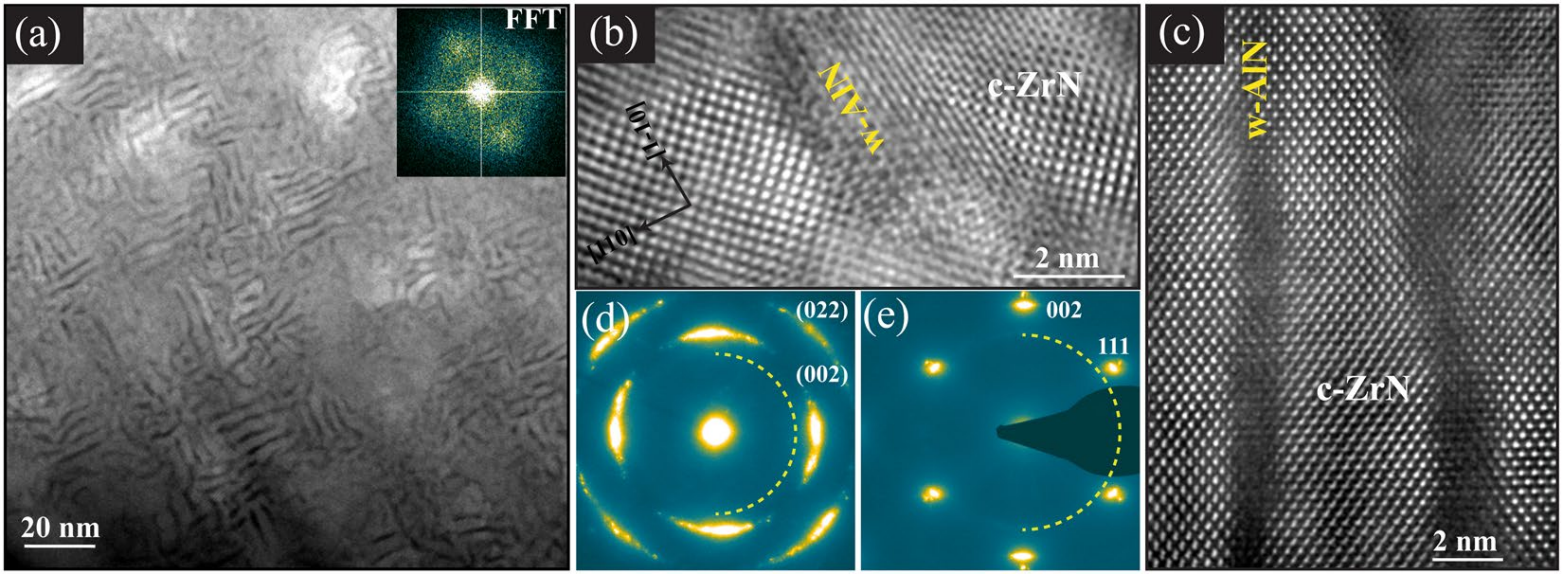
Plan-view (**a**,**b**,**d**) and cross-sectional (**c**,**e**) TEM images of Zr_0.80_Al_0.20_N_0.96_/MgO(001) film deposited at 800 °C. (**a**,**b**) Overview and high resolution HAADF-STEM images with magnified FFT around (000) reflection. (**c**) HAADF-STEM image, (**d**,**e**) SAED patterns. The plan view and cross-sectional images are recorded along ZrN[001] and ZrN[011] zone axes, respectively.

That c-AlN is unlikely to form in decomposition processes in Zr_1−x_Al_x_N alloys is confirmed by investigating Zr_0.80_Al_0.20_N_0.96_/MgO(001) film in *Series-1* deposited at 800 °C. This film with Al content as low as x = 0.2 does not show peak shift in XRD towards lower or higher 2θ angles, corresponding to any in-plane compression or solid solution, respectively. Instead, long range ordered nanolabyrinth structure very similar to 900 °C film form in this film as shown in plan-view HAADF-STEM image and corresponding FFT in Fig. 6a. High-resolution STEM image in Fig. 6b confirms topotaxial relationship as was obsereved for x = 0.31 films. The plan-view images and image recorded in cross-sectional view along ZrN[011] zone axes in Fig. 6c reveal that ZrN lamellae are segregated by ~1 nm wide AlN rich wurtzite lattice, which probably is too small to generate in-plane strain in ZrN lattice and hence no shift in XRD is observed. The presence of w-AlN is also evident from w-(10–10) rings in SAED patterns obtained along the two orientations.

**Figure 7:**
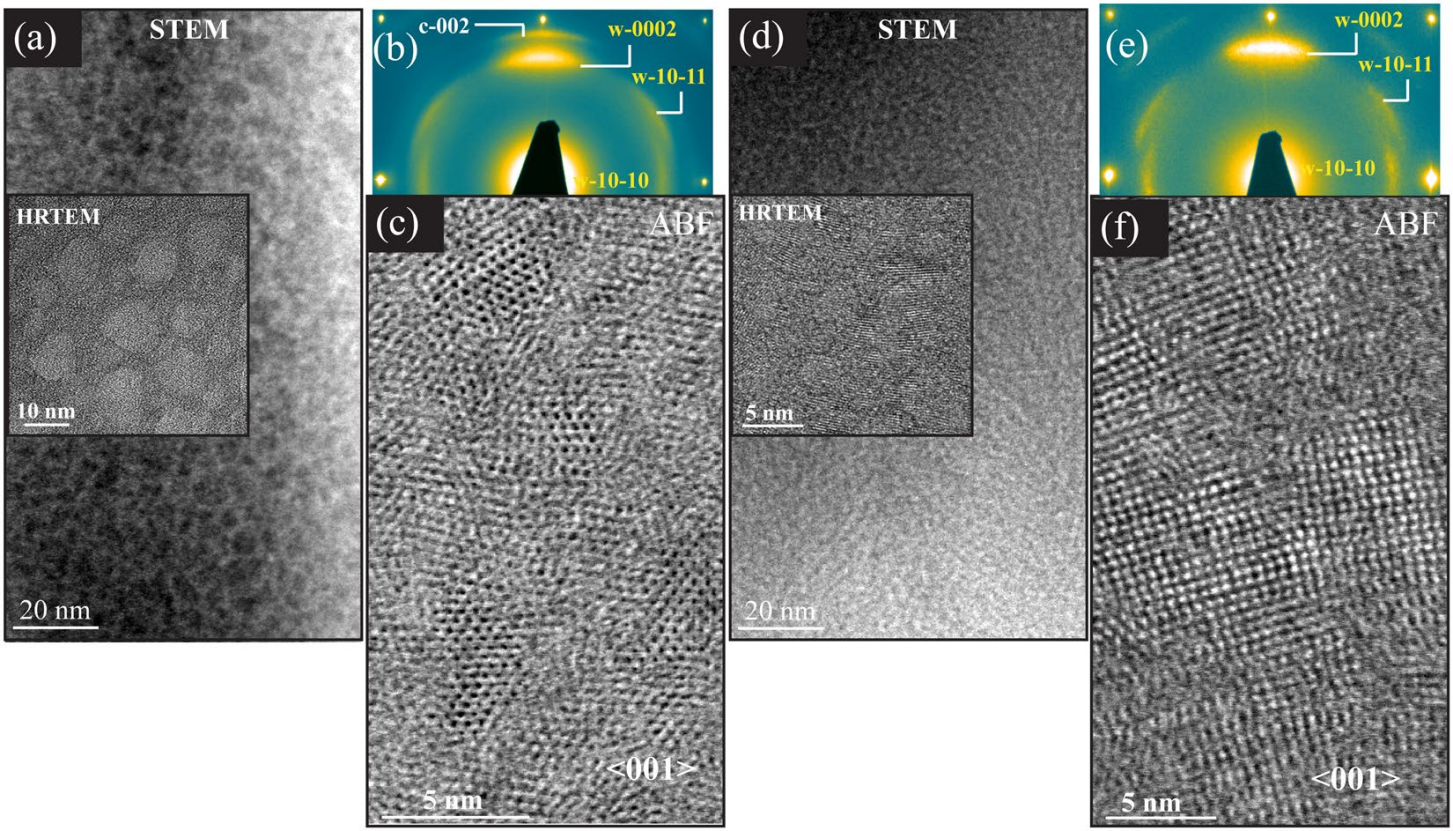
Cross-sectional TEM images of Zr_0.51_Al_0.49_N_1.04_ (**a**–**c**), and Zr_0.25_Al_0.75_N_1.02_ (**d**–**e**) films deposited in *Series-1*: (**a**,**d**) HAADF-STEM, (**b**,**e**) SAED patterns, (**c**,**f**) BF-STEM images.

To investigate structure of the films showing wurtzite peaks in XRD scans (green profiles in Fig. 1), Zr_0.51_Al_0.49_N_1.04_ and Zr_0.25_Al_0.75_N_1.02_ films deposited in *Series-1* are analyzed using TEM, see Fig. 7. It is observed that upon adding Al > 0.49 at.%, the nanostructure morphology completely transforms from ordered lamellas to typical 3D nanocomposites, where equiaxed nanocrystallites of Al-rich phase are embedded in the matrix of Zr-rich phase. However, the crystallography of the respective phases depends on the amount of added Al in the films. The XSTEM and corresponding HRTEM images of x = 0.49 film in Fig. 7a shows two phase composites where dark areas (the contrast is reversed in HRTEM) are Al-rich and bright regions are Zr rich. From the SAED and ABF-STEM image it is evident that Al-rich and Zr-rich domains attain wurtzite and cubic lattice, respectively. For the film with x = 0.75, a very fine composite structure is observed in Fig. 7d. The analysis shows that around these composition and growth temperature an incomplete segregation results in a nanocomposite formation with AlN and Al-rich-ZrN domains with a weak w-AlN <0002> preferential crystallographic orientation in the growth direction. Similar morphology and electron diffraction as of x = 0.75 was recorded for Zr_0.43_Al_0.57_N film deposited at 700 °C in identical conditions as are used in this work (see Fig. 3 in^30^). Combining this information with present XRD analysis, we assume that for x > 0.55 films exhibit identical wurtzite nanocomposite morphology with varying d-spacing depending on Al content. In order to understand Zr incorporation in the wurtzite lattice, we performed an XPS analysis on the films.

### Chemical bonding: XPS spectra analysis

**Figure 8:**
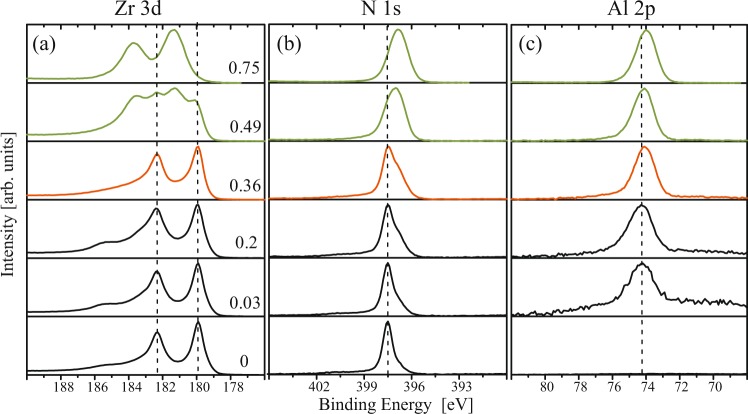
(**a**) N 1 s,(**b**) Al 2p, and (**c**)Zr 3d core level XPS spectra of films deposited on MgO(111) substrate in *Series-1*. see Fig. SM2(a) to relate profiles. Dotted vertical lines are guide markers.

Figure 8 shows Zr 3d, N 1 s, and Al 2p XPS spectra of the films deposited with x = 0 to 0.75 in *Series-1*. The Zr 3d spectra of x = 0–0.36 films exhibit Zr 3d_5/2_ and Zr 3d_3/2_ peaks at 179.9 and 182.3 eV, respectively, corresponding to characteristic stoichiometric c-ZrN bonding^31,32^. A low intensity broad peak centered at 185.5 eV shows up in few films and corresponds to the Zr 3d_3/2_ component due to surface oxide^33^. For x = 0.49 films the Zr 3d spectrum changes abruptly: in addition to the spin-split doublet observed for x < 0.49 films, a doublet shifted by 1.4 eV towards higher binding energy appears, indicating that Zr atoms are present in two distinctly different chemical states The appearance of these new features in the Zr 3d spectra is associated with a complete change of the film nanostructure as revealed by TEM results presented above from ordered lamellas to 3D nanocomposites, with Al-rich wurtzite-structure nanocrystallites embedded in the matrix of cubic Zr-rich phase. Therefore, it seems natural to assign the high-binding energy Zr 3d doublet to Zr atoms incorporated in the wurtzite phase. This interpretation is further supported by the Zr 3d spectrum acquired form the x = 0.75 film in which case the original peaks disappear completely while high-BE peaks remain, indicating that all Zr is embedded in the wurtzite phase. Analogical evolution is observed in the N 1 s spectrum which shows a single narrow peak at a binding energy of 397.5 eV in x = 0 film, corresponding to the chemical state of N in stoichiometric ZrN^32^. Thereafter, upon adding Al an additional shoulder appears in N 1 s peak at lower binding energy, which subsequently grows and widens the peak width up till x = 0.36. For x = 0.49 and 0.75 films the original peak completely disappears and the only single wide peak centered at 397 and 396.85 eV, respectively, are observed. No noticeable trend in the Al 2p spectra is detected except for a subsequent shift in total of 0.2 eV towards lower binding energy (74 eV) upon adding up to 75 at.% Al cations. Overall the BE of 74 eV corresponds very well to 74.1 eV reported for AlN measured on the same instrument^32^.

No change is observed in Zr 3d spectra for Zr_0.69_Al_0.31_N films grown at different temperatures in *Series-2* (see. Fig. SM3 in supplementary material). Similar to Zr_0.64_Al_0.36_N film grown at 800 °C, N 1 s spectra can be convoluted into two peaks where the intensity of the lower energy subpeak seems to be decreasing with increasing temperature. Al-2p spectra are identical for all temperatures except higher oxygen containing 900 °C film, where Al-2p peak shows a chemical shift of 0.4 eV towards higher binding energies.

### Phase stability and decomposition behaviours from first principles

**Figure 9:**
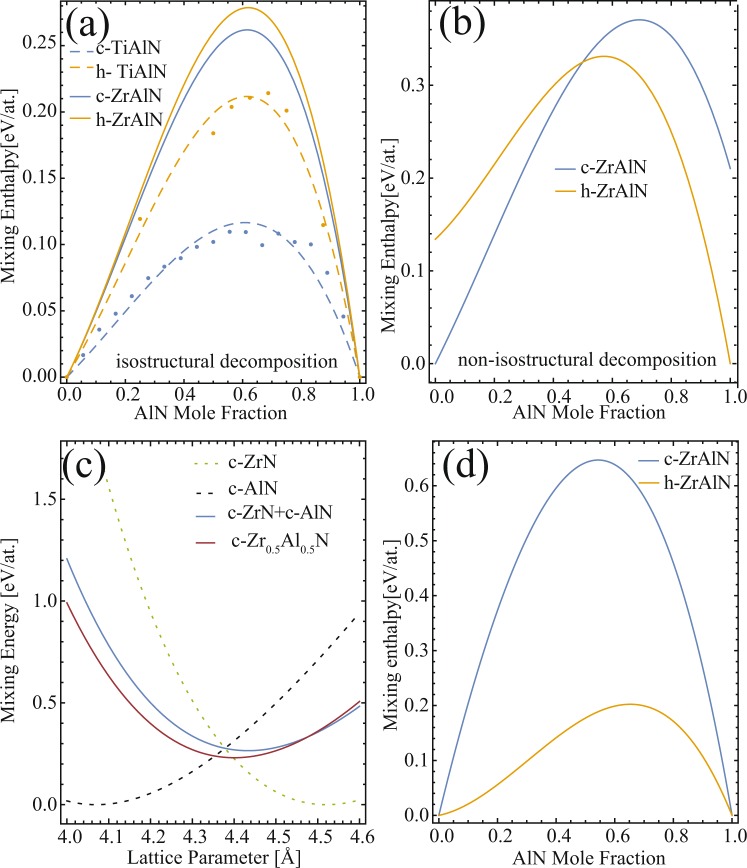
(**a**) Isostructural enthalpy of mixing of cubic(blue) and hexagonal(brown) phases for ZrAlN (solid) and TiAlN (dashed) (**b**) non-isostructural enthalpy of mixing, (**c**) energy-lattice spacing calculations for c-Zr_0.5_Al_0.5_N(red) and a hypothetical isotructural phase separation [in blue according to ref.^4^, (**d**). Isostructural decomposition at fixed pressure of the decomposition products corresponding to an overall energy minimum scenario at a volume fixed to that of the parent phase.

First principle calculation in Fig. 9(a) shows that the driving force for isostructural decomposition is similar for cubic (B1-NaCl) and hexagonal phase ZrAlN, and much higher than for TiAlN for both phases, where additionally the mixing enthalpy of the cubic phase is a factor of ~2 larger than for the hexagonal phase. Moreover, according to^6^ the B4 phase is stable for *x* > 0.4; therefore, *H*_mix_ for the wurtzite phase is generally larger than for cubic phase with predicted metastability range up to *x* ~ 0.4. The driving force for non-isostructural decomposition into the thermodynamically stable w-AlN and c-ZrN, phases [Fig. 9(b)], on the other hand, is higher for the c-ZrAlN compared to the wurtzite one for high Al contents, suggesting a thermodynamic stabilization of the wurtzite phase with respect to the cubic one for these alloys.

In order to understand the non-isostructural segregation in the cubic-phase films, mixing energy vs. lattice spacing is calculated in Fig. 9(c) using the approach described in refs^4,6^. Here, the energy of a solid solution is compared with the energy of isostructural decomposition products (cubic phases) assuming the same specific volume (and hence the same cubic lattice constant) as the parent ternary phase: that is, all energies on the right-hand side of Eq. (1) are evaluated at the same volume corresponding to the volume of the solid solution. The energy of c- Zr_0.5_Al_0.5_N as a solid solution (red line) is lower than that of c-AlN and c-ZrN (blue line) strained to the same (cubic) lattice constant for lattice spacings less than 4.5 Å, i.e., for a very broad range of possible stress states of the quasi-binary ZrN-AlN (calculated equilibrium lattice constant of ZrN is 4.618 Å an predicted lattice constants of c-AlN is 4.069 Å^34^). Hence, due to volume mismatch of respective cubic binaries, the decomposition is not expected to proceed spinodally, thus similar to the ScAlN case described in^4^.

Nevertheless, the assumption of equal specific volumes implies different pressures in each phase, and hence not fulfilling a mechanical equilibrium criterion in the decomposed state. For that reason, another evaluation of the energy differences was performed, in which the pressure (instead of specific volume) in both decomposed phases is equal. The enthalpy of mixing now contains additional term, +*pV*, which was added to the energies of the binary AlN and ZrN phases on the right-hand side of Eq. (1). It is worth noting that this scenario corresponds to an overall energy minimization of the decomposed states with a constraint that the total volume of the decomposition products matches that of the parent phase. This is, however, reasonable as no voids are needed or observed as a consequence of the decomposition, and in the initial stages of the decomposition process, the surrounding parent matrix would guarantee no volume changes in the region where decomposition takes place. The corresponding results, plotted in Fig. 9(d), yield H^hex^_mix_ < H^cub^_mix_, i.e., the wurtzite phase becomes significantly stabilized with respect to the cubic phase, in agreement with the experimental observations.

### Nanoindentation response versus film composition and T_s_

**Figure 10:**
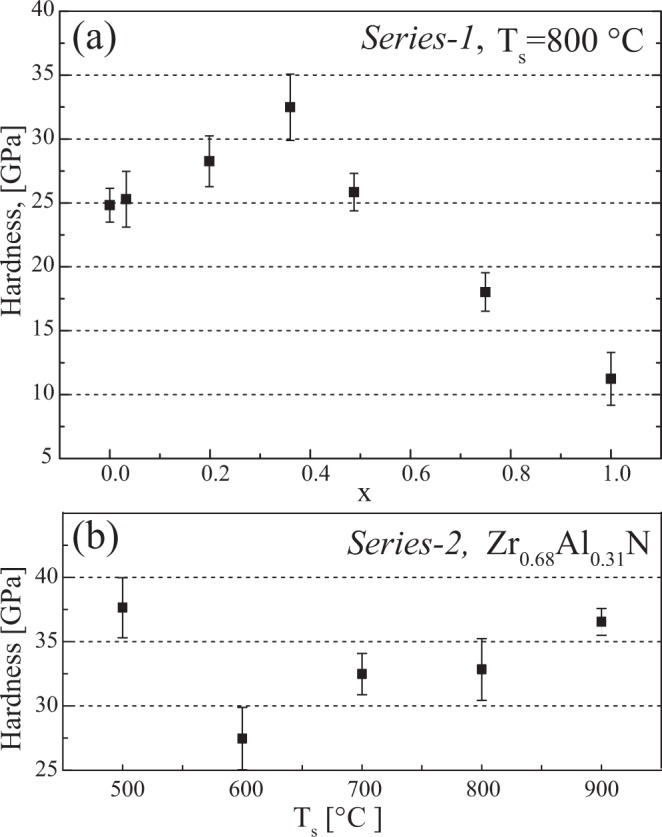
Nanoindentation hardness of: (**a**) *Series-1* corresponding to the films shown in Fig. 1(a), (**b**) *Series-2* corresponding to the films shown in Fig. 3a.

Figure 10(a) shows the nanoindentation hardness of seven films deposited in *Series-1* on MgO(001) substrate with varying composition. The hardness of 25 ± 1 GPa of high-quality single-crystal ZrN film is on the same scale as has been reported for group IV transition metal binary nitrides, i.e., for TiN and HfN sputtered films on MgO^35,36^. Incorporation of Al results in a gradual enhancement in hardness up to maximum value of 33 GPa for Zr_0.64_Al_0.36_N_0.97_. The film with the highest nanoindentation hardness possesses two phase nanolabyrinth lamellar structure when grown between 700–900 °C. Figure 10(b) shows the hardness of Zr_0.69_Al_0.31_N films grown at varying T_s_ in *Series-2*. Here, the highest hardness of 37 GPa is measured for single cubic phase film grown at 500 °C. Hardness drop to about 27 GPa at 600 °C, and gradually increases till 36.5 GPa for the films grown at 900 °C.

For high Al content films in *Series-1*, the hardness drops to 26 and 18 GPa on further increase in Al to 49 and 75 at%, respectively, where a two-phase nanocomposite is formed in the films.

**Figure 11:**
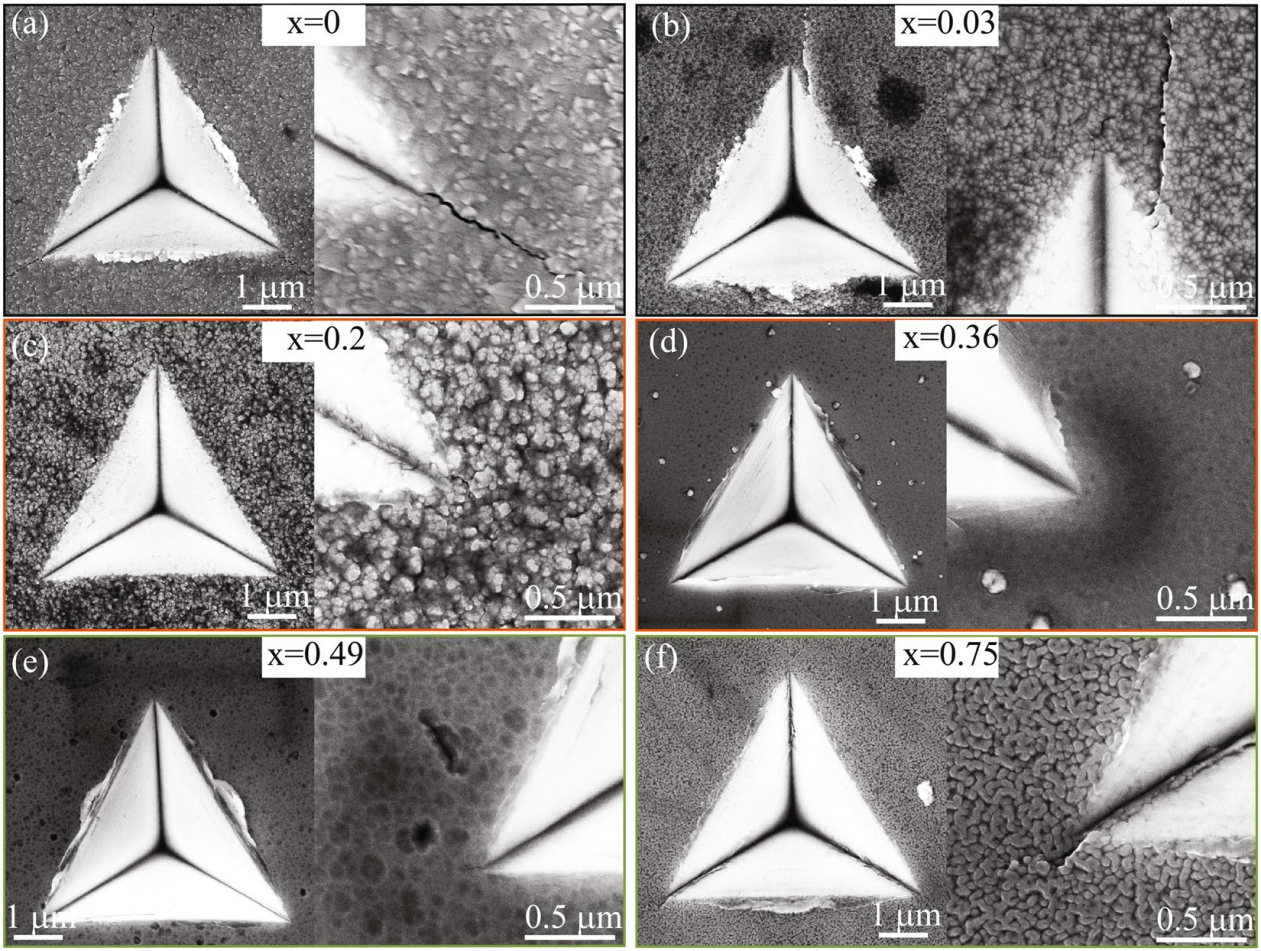
SEM images (overview and magnified at an impression corner) of the indentation induced cracking at a force of 200 mN for x = 0–0.75 films deposited on MgO(001) in *Series-1*.

Figure 11(a–f) shows overview and magnified SEM images of the indentation induced cracking at a force of 200 mN for x = 0–0.75 films deposited in *Series-1*. Since the indentation geometry is identical and films were about equally thick, the morphology of indents and formation of radial cracks reflects the fracture toughness of the films. For x = 0 and 0.03 films, μm long radial cracks were observed at the corners of the indent. For the films having nanolabyrinthine morphology, see Fig. 11(c,d), no radial crack emanated from the impression corner, particularly for x = 0.36 film. For x = 0.45 and 0.75 nanocomposites films the length of crack limited to ~0.1 μm. For x > 0.2 films, where no or very small radial cracks generated, picture-frame cracks inside the indented surface were observed. Assuming sharp geometry of the indenter, the most likely explanation of picture-frame crack generation is the deformation of substrate which yields before the thin hard (x = 0.2 and 0.36) and tough (x = 0.45 and 0.75) film, adding bending influence on the deformation of film^37^. In short, a high fracture resistance (based on radial crack length and density) observed for both, the semi-coherent labyrinthine and incoherent nanocomposite coatings, suggests that different deformation mechanisms are active in the two types of self-assembled nanostructures.

## Discussion

### Influence of substrate on the film growth

In terms of phase formation and microstructure evolution with increasing AlN in *Series-1*, films exhibit overall similar features for single-crystal and polycrystalline substrates. A strong competitive growth of 002 texture over 111 occurs upon adding AlN in the dominant cubic phase films i.e., with x = 0, 0.03, and 0.2. Since, no indication of a lattice parameter decrease due to the Al substitution in the ZrN lattice is observed in these cubic phases containing films, it is evident that at 800 °C the solid solubility limit is much lower than what is usually obtained for low-temperature-grown films. From the XRD and TEM analysis, it is clear that the formation of coherent cubic/wurtzite lamellae in x = 0.2, 0.36 films, noncoherent cubic/wurtzite domains in x = 0.49 film, and isostructural decomposition in wurtzite phase containing x > 0.55 films, is inherent to the growth kinetics where substrate plays a minor role. This also implies that the process can be transferred to any industrially relevant substrate with retained nanolabyrinthine composite film structure.

### Self-assembly into nanolabyrinthine lamellar structure

During the initial decomposition stage of Ti_1−x_Al_x_N alloys, isostructural TiN and AlN rich domains self-assemble along elastic complaint crystal directions (which are <100> for x > 0.28^38,39^) and the resulting assembly resembles a *checkboard* pattern^2,3^. The similar pattern is observed in plan-view TEM images by Abidi *et al*. during magnetron sputter deposition of Ti_0.5_Al_0.5_N/MgO(001) films when grown between 540–560 °C^1^. The difference in the later was the lamellar extension only along the [001] growth direction, which indicates that the composition modulation was initiated at the surface during deposition rather than in the bulk. Depending on the AlN content and decomposition stage (in temperature or time) the compositional modulation wavelength of coherent domains in Ti_1−x_Al_x_N alloys is measured from 2.3 nm^1^ to 10 nm^40^, i.e., c-AlN rich domains can be as wide as ~5 nm. To a certain extent, the self-assembly of nanolabyrinthine lamellar structure in the present work is comparable to the formation of checkboard pattern observed in ref.^1^.

Here, the orientation of basal planes of the w-AlN lamellae solely along the [001]_ZrN_ growth direction suggests that the lamellae form during the growth of the film, as if this was not the case the cubic symmetry of the ZrN-phase would allow for lamellae to form along the [100] and [010] directions as well^4^. The film/substrate interface is investigated in atom probe tomography (APT) of Zr_0.69_Al_0.31_N (800 °C) film in^29^ and it shows the onset of lamellar growth occurs by surface nucleation, 5–8 nm above the MgO substrate. Below ~6 nm, ZrN and AlN have no site preference when condensed on the substrate and they randomly nucleate on the cubic growth front. However, during deposition of each successive atomic layer, surface segregation (due to enhanced immiscibility at high growth temperature) introduces variation in the energy of surface atoms depending on the chemical identity and composition of the underlying layers. During steady-state growth, AlN nucleates into equilibrium wurtzite crystal structure, which has only mirror symmetry with regards to the cubic template and matrix, leading to strong (semi)coherency strain. This results in <110> directions coincide with the nucleation of wurtzite domains. The in-plane shape of the lamellae, i.e., elongated AlN, with a long (semi)coherent edge $[(110)ZrN//(11\bar{2}0)AlN]$ and a short incoherent one is a strong indicator for their growth being directly in the wurtzite phase.

The segregation of AlN directly into the wurtzite lattice when deposited above 600 °C and with AlN content ≥20 at.% (see Figs 2, 4, 6 and 7) in Zr_1−x_Al_x_N is the most striking difference compared to Ti_1−x_Al_x_N alloys. It is a general perception that AlN domains should be large enough to make the cubic-to-wurtzite transformation energetically favorable. However, the presence of w-AlN in nanolabyrinthine form in Zr_0.69_Al_0.31_N_0.97_[x = 0.31, T_s_ = 700 °C; Fig. 5(c)] and Zr_0.80_Al_0.20_N_0.96_[x = 0.2, T_s_ = 800 °C; Fig. 6] films clearly shows that AlN directly form wurtzite lattice regardless of the domain size. The possible explanation is the staggering volume mismatch (9% misfit) of the respective cubic binaries averting the decomposition to proceed spinodally. The lower energy calculated for a solid solution c-Zr_0.5_Al_0.5_N compared to c-AlN and c-ZrN strained to the same (cubic) lattice constant [see Fig. 9(c)] support the non-isostructural decomposition in the present alloys.

An apparent signature of w-AlN formation in nanolabyrinthine films is an in-plane compression of c-ZrN lamellar lattice resulting in larger out-of-plane lattice parameter [see Figs 1 (d) and 3(c)]. However, at lower x and T_s_ the domain size of w-AlN is too small to exert noticeable compression in the c-ZrN lattice and hence no lattice parameter increase is observed.

The increase in elemental segregation and domain size with growth temperature is apparent when compared between 700 and 800 °C films (see Fig. 5) and thus tallies with a surface-diffusion-driven segregation. Further increase in growth temperature that is from 800 to 900 °C results in more ordered and pure domains, but domains size does not scale with temperature. Thus, one can conclude from Fig. 5 that long-range order of nanolabyrinthine structure improves upon increasing temperature from 800 to 900 °C, however careful analysis of FFT´s of STEM images reveal higher order in 700 °C grown film. Similarly, higher long-range order identical to Zr_0.69_Al_0.31_N_0.97_ (900 °C) nanostructure is observed in less AlN-containing Zr_0.8_Al_0.21_N_0.96_ (800 °C, see- Fig. 6). From these observations, we conclude that long-range order of labyrinthine is more related to the overall epitaxy of ZrN domains, which become better in crystal quality with decreasing AlN content and with increasing T_s_ from 800 to 900 °C.

We conclude that similar to spontaneous ordering in two phase oxide nanocomposite epitaxial thin films^41^, both horizontal and vertical epitaxy plays a role and minimization of elastic strain energy and interfacial energy leads to formations of nanolabyrinthine. The vertical lattice mismatch between nanocolumns provide mechanical strength that goes substantially beyond what is possible in lateral naolaminated structures

### Solubility limit of Al in fcc-ZrN

The Al fraction where phase transformation from fcc to wurtzite Zr_1−x_Al_x_N phase occurs, defines the effective limit of AlN solubility in fcc-ZrN and is shown to be ~x = 0.2 by experimental observation for the films grown here >700 °C. This critical fraction is much less than reported in^6,8,10^. The reason for the range of AlN contents for phase separation is likely difference in measurement accuracy, where the present study is the most sensitive.

Moreover, Sheng *et al*. calculates temperature dependence of solubility limit and finds increase in Al fraction into fcc lattice from x = 0.47 (T = −273 °C) to x = 0.57 (T = 1000 °C)^10^. Contrarily, in high-temperature growth, we observe segregated c-ZrN and w-AlN domains at very low Al content i.e., in x ≥ 0.2 films grown at temperatures above 700 °C, indicating decreased solubility at high temperatures. The results are more similar to CrN–AlN system, where Al fraction is calculated to be shifted strongly to lower values upon increasing temperature^42^. This apparent contradiction can be traced to the fact that the previous works on ZrAlN [6, 10] considered only isostructural decomposition scenario, while such spinodal decomposition is suppressed in the present case, as discussed above.

### Solubility of Zr in w-AlN and iso-structural decomposition in w-ZrAlN

Our findings in x = 0.49 and x = 0.75 films agree with calculations and experimental reports showing decomposition in w- Zr_1−x_Al_x_N^8,13^. The larger lattice parameter of segregated w-ZrAlN rich domains in dual phase x = 0.49 film, and extremely fine domains in x = 0.75 film, both indicate quenched diffusion of Zr in the wurtzite lattice, similar to what is observed for Y in metastable w-Y_0.13_Al_0.87_N films grown at 900 °C^43^. The main advantage of slow diffusion/coarsening of the nanodomains is thermal stability of wurtzite crystal structure, as is shown in 1100 °C annealed cathodic arc evaporated w-Zr_1−x_Al_x_N films^13^. Here we address prominent features of these wurtzite-phase-based films.

#### Iso-structural decomposition in w-ZrAlN

During surface-diffusion-initiated segregation w-Zr_0.25_Al_0.75_N (800 °C) films attain a regular nanocomposite structure, where w-AlN-rich crystallites are surrounded by distorted wurtzite lattice of Zr-rich domains with compositional modulation of ~5 nm. From the XPS analysis, it is clear that the chemical state of Zr is different to what is expected for fcc-ZrN. Previously, during annealing of cathodic arc evaporated w-Zr_0.3_Al_0.7_N films^13^, nanoscale compositional modulations within the hexagonal ZrAlN lattice in the form of Al-rich and Zr-rich layers are observed. Hence, we conclude that unlike c- Zr_1−x_Al_x_N, coherent spinodal decomposition is probable in w-Zr_1−x_Al_x_N.

#### Stabilization of w-ZrAlN versus c-ZrAlN

A dual-phase cubic and hexagonal mixture up to x ≈ 0.68, and single wurtzite phase for higher AlN mole fraction is predicted to be energetically favorable in Zr_1−x_Al_x_N alloys^6,10^. Also, probability of spinodal decomposition in hexagonal alloys is reflected from the first principle calculations suggesting lower driving force for non-isostructural decomposition in hexagonal phase with respect to the cubic one [see Fig. 9(b)]. Moreover, by considering the overall energy minimization of the decomposed states with the constrained that the total volume of the decomposition products matches that of the parent phase, H^hex^_mix_ is found to be less than H^cub^_mix_, i.e., supporting the stabilization of hexagonal phase. In case any cubic-phase domains were to nucleate in the w- Zr_1−x_Al_x_N alloy, its ~20% smaller molar volume would rapidly lead to a ceased growth as it becomes diffusionally isolated.

#### Formation of metastable w-ZrN

The core-level energy shifts in the XPS spectra (1.4 eV in Zr 3d, and −0.65 eV in N 1 s compared to x = 0.36 film consisting of c-ZrN) in w-Zr_0.25_Al_0.75_N film indicate an increased negative charge transfer from Zr to N and Al atoms, hence more of ionic bond character. This was also predicted in band-structure calculations indicating w-ZrN formation energetically unfavorable^44^. Experimental findings of w-ZrAlN in this work is an important result as it opens the possibility of formation of metastable zincblende/wurtzite structures of ZrN with group-IIIA–N alloys, in particular Zr_1− x_In_x_N alloys, as the interatomic distances of ZrN and InN in the zincblende and wurtzite structures are very close^45^. Moreover, further understanding of electronic structure of w-Zr_1− x_In_x_N will also help to underline the mechanisms behind alleged slow diffusion in these films which has direct impact on extreme thermal stability and mechanical integrity during high-temperature operations^13,30,46^.

### Hardness variations with composition and temperature

Despite the high growth temperature used in the present work, the overall increase in hardness with increasing Al content up to x ≈ 0.4 in Zr_1−x_Al_x_N alloys is in agreement with previous reports^8,47^. The significance of optimum composition is evident from the Zr_0.69_Al_0.31_N films, all having high hardness values but significantly different morphology and nanostructures when deposited over a wide temperature range, see Fig. 10(b). Based on strengthening mechanisms we can discuss three different categories of the present films.

#### Solid solution strengthening in low temperature grown Zr_0.64_Al_0.31_N

In analogy to the Ti_1−*x*_Al_*x*_N, the highest hardness of 37 GPa measured for single cubic phase film grown at 500 °C is ascribed to solid-solution strengthening and film densification due to ion assisted growth. Mayrhofer *et al*. explain that a low average delivered energy to the growing surface significantly lower the hardness in 500 °C reactively sputtered Zr_0.66_Al_0.34_N films^9^. Therefore, we conclude that the bombardment using low-energy high-flux ions in the present work significantly improves the quality of solid solutions formed in c-Zr_1−x_Al_x_N films. The slight drop in 600 °C grown films is ascribed to highly distorted cubic crystal structure at the onset of segregation.

#### Hardening in the nano-labyrinthine films

Nanoindentation experiments showed that highly ordered nano-labyrinthine Zr_0.64_Al_0.31_N films had a hardness of 36 ± 1 GPa, an increase by more than 40% compared to ZrN (25 ± 1 GPa), and 130% compared to AlN (11 ± 1 GPa). The structural analysis revealed a short-range order of labyrinthine pattern due to fiber texture present in 800 °C and 700 °C Zr_0.64_Al_0.31_N films, which can cause a slight decrease in hardness. The high hardness and high fracture resistance in these structures is ascribed to the combined hindering of dislocation glide by inhomogeneous strain due to the mosaicity in the phases, and the lack of common glide planes in the c-ZrN and w-AlN lamellae. The as-formed (semi) coherent interfaces provide for both Koehler and coherency hardening mechanisms.

#### Two-phase equiaxed nanocomposite mechanical properties

The lack of common glide planes in the c-ZrN and w-AlN lamellae cannot be the exclusive reason for high hardness and considerable high fracture resistance, observed in Zr_0.51_Al_0.49_N film having incoherent interfaces between w-AlN nanocrystatlites and c-ZrN rich matrix. Thus, impeded dislocation motion due to grain boundaries (Hall-petch strengthening) is most likely the active mechanism in these films. Upon further increase in x, nanocomposite attains coherent interfaces within wurtzite lattice and the hardness drop to ~18 G Pa, is similar to what is expected in other w-transition metal aluminum nitrides^48^.

## Conclusion

We have shown two routes of self-organization in Zr_1−x_Al_x_N alloys resulting in highly regular crystalline nanocomposite structures, upon varying composition during magnetron sputtering of ZrAlN alloys. Under the identical process conditions including a high growth temperature providing sufficient surface mobility to add atoms, low AlN (x < 0.36) containing films decompose into nearly pure c-ZrN and w-AlN lamella forming highly ordered nanolabyrinthine structure, whereas high AlN content results in 3D-nanosrcuture of w-AlN nanocrystallites embedded in w-ZrAlN matrix. No experimental observation of formation of metastable c-AlN, at all x and T_s_, suggests that non-isostructural decomposition is the likely, energetically more favorable, mechanism in Zr_1−x_Al_x_N alloys. The associated hardness increase in the alloys upon decomposition is manifested to induced strain upon (semi)coherent-interface formation between c-ZrN and w-AlN. The first principle calculations resulting in lower energy for a solid solution c-Zr_0.5_Al_0.5_N than c-AlN and c-ZrN domains strengthen our experimental findings of non-isostructural decomposition in x < 0.5 alloys.

Uunlike c-Zr_1−x_Al_x_N, coherent spinodal decomposition is probable in w-Zr_1−x_Al_x_N. This was experimentally observed in x = 0.75 film and is also reflected from the first principle calculations suggesting lower driving force for non-isostructural decomposition in hexagonal phase with respect to the cubic one. Two interlaced staggering findings of w-Zr_1−x_Al_x_N alloys are: (a) the core-level energy shift in Zr 3d XPS spectra which signifies the novel prospect of formation of metastable zincblende/wurtzite structures of ZrN with group-IIIA–N alloys, and (b) the quenched diffusion of Zr in the wurtzite lattice, providing high structural stability of w-Zr_1−x_Al_x_N alloys at elevated temperatures up to 900 °C.

The two types of nanostructures are shown to exhibit high hardness and high fracture resistance ascribed either due to the hindering of dislocation glide in the c-ZrN and w-AlN lamellae forming (semi)coherent interfaces and impede dislocation motion due to grain boundaries in incoherent 3D- equiaxed nanocomposites. Finally, the formation of w-AlN domains “in as-deposited films in the composition range 0.2 < x < 0.75” suggests that Zr_1−x_Al_x_N nanocomposites are exclusive coating materials system among the other transition metal aluminum nitrides which can be used during high temperature operations without structural and mechanical degradation of the alloys.

## Electronic supplementary material

Supplementary material
