# Supplementary material for: Self-structuring in Zr1−xAlxN films as a function of composition and growth temperature
Source: Sci Rep. 2018 Nov 5;8:16327. doi: 10.1038/s41598-018-34279-w (PMC6218527; doi:10.1038/s41598-018-34279-w)
Supplement: Supplementary file 1 — Supplementary material [file 41598_2018_34279_MOESM1_ESM.docx]

# Self-structuring in Zr_1-x_Al_x_N films as a function of composition and growth temperature

N. Ghafoor, I. Petrov, D. Holec, G. Greczynski , J. [Palisaitis](http://www.ep.liu.se/PubList/Default.aspx?userid=juspa01), P.O.A Persson, L. Hultman, J. Birch

**Supplementary Material**


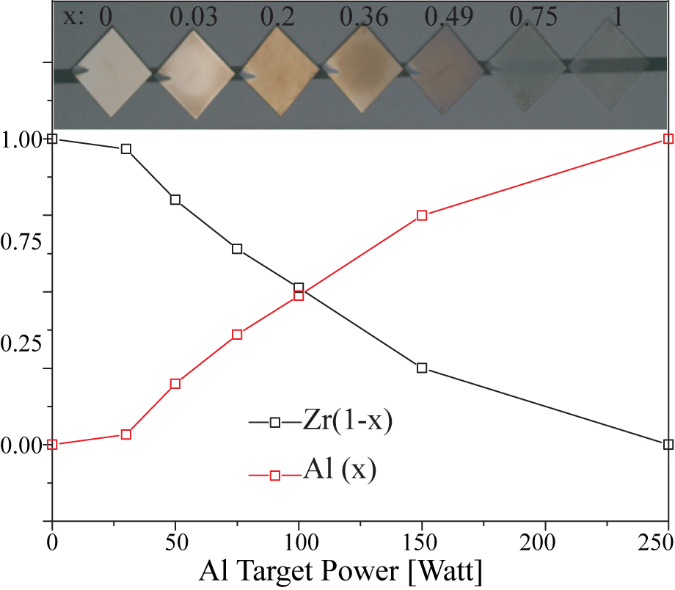


Fig. SM1. Zr and Al content plotted against Al-target power (p_Zr_+p_Al_ = 250 W) for the films deposited in *Series-1*. The color change in the films with corresponding composition is shown in the inserted optical micrograph.

Fig. SM1 illustrates how the metal atom ratio in the films varies with target power, here plotted for Al target. The crossover at 100 W indicates slightly higher sputter rate of Al compared to Zr at a given power. No anomalies in nitrogen distribution with increasing Al target power were observed except at 250 W where under stoichiometric AlN_0.9_ film contained 3 at.% of oxygen (see main text). Also inserted in Fig. SM1 is the optical photograph of the corresponding 1.5 μm thick films on MgO(001) substrates deposited in *series-1* at 800° C (see the description in the main text).


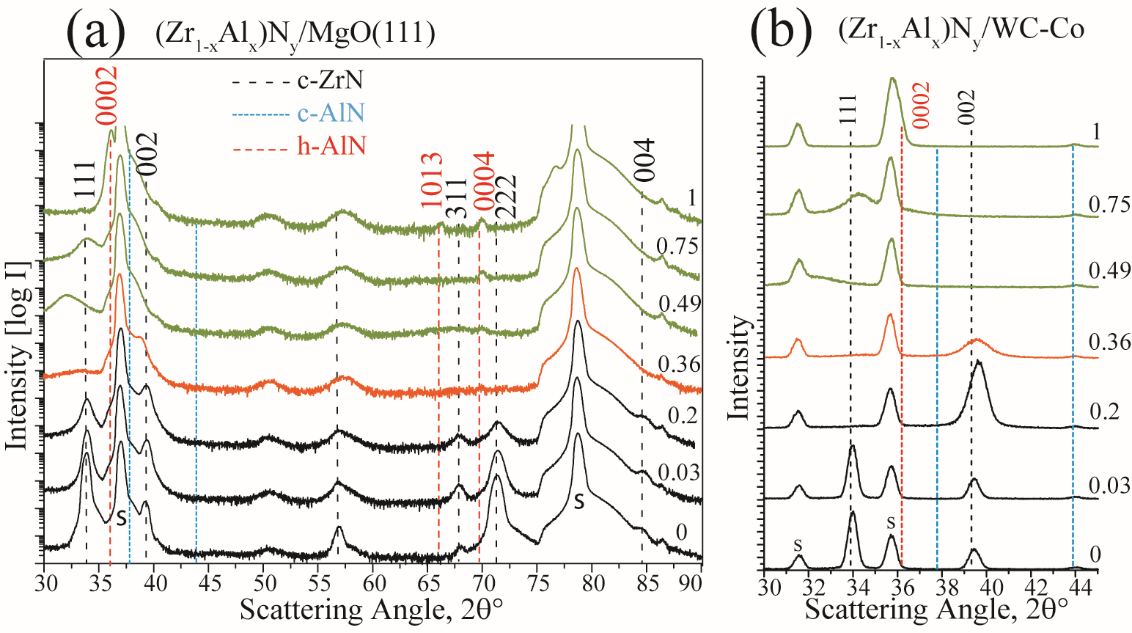


Fig. SM2. ω−2θ X-ray scans of Zr_1-x_Al_x_N_y_ (0≤ x ≤ 1) films deposited in *Series-1*; (a) on MgO(111), and (b) WC-Co substrates. For clarity, the scans are offset vertically in each graph and labelled according to Al content, x, in the films. The dotted lines are marked at standard c- ZrN (black), c-AlN (blue), and h- AlN(red) lattice reflections.

Fig. SM2(a) shows that similar to MgO(001), the films with x=0, 0.03, and 0.2 also exhibit peaks originating from the single cubic phase when grown on MgO(111) substrate. However, due to the epitaxy, the ZrN(x=0) film has strong (111) texture, which becomes weaker upon adding 3 and 20 at.% Al. For x=0.36, the only visible film peak is cubic 002 which shifts to lower angles as in the case of MgO(001). Compared to corresponding films grown on MgO(001), the noticeable differences in w-0002 reflection in MgO(111) grown films with high Al content (x=0.49, 0.75, and 1) are, higher intensity, smaller FWHM, and slightly smaller 2θ angle in x≠1 films. The latter can be a consequence of a higher substitution of Zr in the wurtzite lattice. The XRD of films on polycrystalline WC-Co substrates is shown in Fig. SM21(b). Here, it is clear that in the absence of the epitaxial force of single-crystal substrate, (111) texture is the dominant one in ZrN(x=0) film and the preferential orientation completely switches from (111) to (002) upon adding 20 at.% Al. High growth temperatures and high-flux low-energy ion bombardment used in the present sputtering process may also influence this strong texture modifications, as no such trend is seen when films are sputter deposited at 100 °C on WC-Co substrate ^8^. Unlike on MgO substrates, a slight shift of 0.20° towards higher 2θ in x=0.2 film, and no dominant shift towards lower angles in x=0.36 film is observed for 002 peak. For further details see the main text.


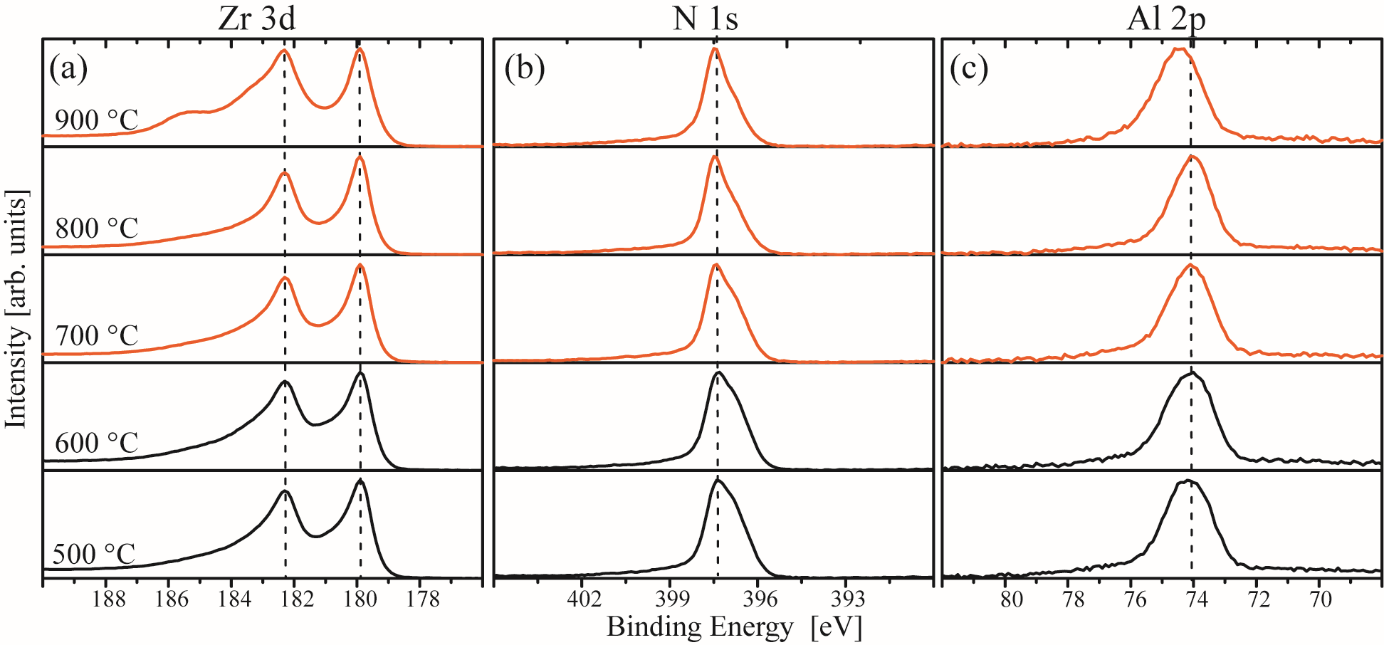


Fig. SM3. (a) N 1s,(b) Al 2p, and (c)Zr 3d core level XPS spectra of films deposited on MgO(001) substrate in series-2. see Fig 6(a) to relate profiles. Dotted vertical lines are guide markers(see the main text).
